# 

**Published:** 1904-01

**Authors:** 


### CONTENTS.

ORIGINAL COMMUNICATIONS—

A PHYSIOLOGICAL STUDY OF TOBACCO AND ITS ACTION UPON THE ORGANISM. By Dr. Georges Petit.... 649
THE QUESTION OF NASAL TREATMENT FOR THE CURE OF DISEASES OF THE EAR. By Alex. W. Stirling, M.D , C.M. (Edin.); D.P.H. (Lond.), Atlanta, Ga.... 656
COMBINED ANESTHESIA WITH ETHYL CHLORIDE AND ETHER. By William Pebrin Nicholson, M D , Atlanta, Ga.... 660

EDITORIAL NOTES AND COMMENTS—

SOMEWHAT EGOTISTICAL.... 664
THE SOUTHERN SURGICAL AND GYNECOLOGICAL ASSOCIATION.... 665
EDITORIAL BREVITIES.... 669

NEWS AND NOTES.... 670

CONTENTS CONTINUED.... X

